# 大体积直接进样-超高效液相色谱-三重四极杆质谱法测定水中7大类42种抗生素残留

**DOI:** 10.3724/SP.J.1123.2021.08010

**Published:** 2022-04-08

**Authors:** Huijing SUN, Peiwen LI, Beibei ZHANG, Huiming CHEN

**Affiliations:** 1.江苏省环境监测中心, 国家环境保护地表水环境有机污染物监测分析重点实验室, 江苏 南京 210019; 1. State Environmental Protection Key Laboratory of Monitoring and Analysis for Organic Pollutants in Surface Water, Jiangsu Provincial Environmental Monitoring Center, Nanjing 210019, China; 2.上海爱博才思分析仪器贸易有限公司, 上海 200335; 2. SCIEX Analytical Instrument Trading Co, Shanghai 200335, China

**Keywords:** 大体积直接进样, 超高效液相色谱-三重四极杆质谱, 抗生素, 水体, large volume direct injection, ultra high performance liquid chromatography-tandem mass spectrometry (UHPLC-MS/MS), antibiotics, water body

## Abstract

抗生素作为新型有机污染物在自然水体中被频繁检出,检出种类多且含量水平低,为了实现更加快速、全面、准确的高通量分析,研究开发了一种利用大体积直接进样测定水中7大类(磺胺类、林可酰胺类、喹诺酮类、大环内酯类、四环素类、头孢类及氯霉素类)42种抗生素的超高效液相色谱-三重四极杆质谱法。水样经0.22 μm滤膜过滤,加入Na_2_EDTA并调节pH值至6.0~8.0,加入内标混匀后,采用Phenomenex Kinetex C18柱(50 mm×30 mm, 2.6 μm),以0.1%(v/v)甲酸水溶液-乙腈作为流动相进行梯度洗脱,质谱智能化分时间段-多反应选择离子监测(Schedule-MRM)模式进行检测。42种抗生素在相关线性范围内线性良好(*r*=0.9949~0.9995),回收率为80.1%~125%,相对标准偏差为0.8%~12.2%,方法检出限为0.015~3.561 ng/L。将该方法应用于10份水源水和5份末梢水的检测,结果显示在42种抗生素中,12种抗生素有检出,包括磺胺类、大环内酯类、林可酰胺类和氯霉素类,其在水源水中的检出率达100%;林可霉素和氯霉素是检出质量浓度最高的两种抗生素,它们的质量浓度范围分别为3.83~13.8和24.8~33.6 ng/L。该方法从检出限和回收率两方面与标准方法和文献报道进行了比较,检出限及回收率均满足要求。该方法与传统前处理方法相比具有简单、快速、绿色、精密度高、准确度高、消耗样品量小的优点,能用于地表水、地下水、末梢水等较为洁净水体中42种痕量水平的抗生素测定。

## Abstract (en)

Antibiotics are emerging contaminants that have recently attracted attention. They have been detected in natural water and pose health concerns owing to potential antibiotic resistance. Antibiotics are ubiquitous in aquatic environments, with a wide spectrum and trace levels. It is difficult to detect all types of antibiotics with completely different physicochemical properties. Solid phase extraction (SPE) is a common sample preparation procedure. For a fast and high-throughput continuous on-line analysis of these emerging contaminants, a method for the determination of 42 antibiotics (grouped into seven categories: sulfonamides, fluoroquinolones, lincosamides, macrolides, tetracyclines, cephalosporins, and chloramphenicols) in environmental water was developed based on ultra high performance liquid chromatography combined with tandem mass spectrometry (UHPLC-MS/MS) involving large volume direct injection without sample enrichment and cleanup.

The collected water samples were filtered through a 0.22-μm filter membrane, their pH levels were adjusted to 6.0-8.0 after adding Na_2_EDTA, and then the solutions were mixed with an internal standard. The addition of Na_2_EDTA contributed to the release of tetracyclines and fluoroquinolones from the metal chelate. Improved recoveries were observed for all the compounds when the pH of the aqueous solution was set at 6.0-8.0. The optimized UHPLC conditions were as follows: chromatographic column, Phenomenex Kinetex C18 column (50 mm×30 mm, 2.6 μm); mobile phase, acetonitrile and 0.1% (v/v) formic acid aqueous solution; flow rate, 0.4 mL/min; injection volume, 100 μL. In the UHPLC-MS/MS experiment, chloramphenicol, thiamphenicol, and florfenicol were analyzed in the negative ionization scheduled multiple reaction monitoring mode (scheduled-MRM), while the other 39 antibiotics were analyzed in the positive scheduled-MRM mode. This acquisition method improved the response of each target compound by dividing the time of the analysis test cycle and scanning the ion channels of chromatographic peaks at different time periods. The ionspray voltage was set at 5500 and -4500 V in positive and negative modes, respectively. The source temperature for both ionization modes was set at 500 ℃, which was optimized to improve the sensitivity. Instrumental parameters like collision energy and declustering potential were also optimized.

Good linearity was observed for all the tested antibiotics, with a correlation coefficient (*r*) greater than 0.995. The method detection limits (MDLs) were 0.015-3.561 ng/L. The average recoveries ranged from 80.1% to 125%, while the relative standard deviations (RSDs) were between 0.8% and 12.2%. The method was successfully applied to the determination of 10 source water samples and 5 tap water samples. Twelve antibiotics, viz. sulfachloropyridazine, sulfadiazine, sulfamethazine, sulfamethoxazole, sulfisomidine, clindamycin, lincomycin, roxithromycin, clarithromycin, erythromycin, thiamphenicol, and forfenicol, were detected in the 10 water samples with a detection frequency of 100%. The total antibiotic content in each sample ranged from not detected to 80.3 ng/L. Lincosamides and chloramphenicols were the predominant antibiotics in the water samples, with contents in the ranges of 3.83-13.7 and 4.23-33.6 ng/L, respectively. Therefore, the large volume direct injection method exhibited good performance in terms of MDL and recovery compared to standard methods and those reported previously.

Compared with traditional pretreatment methods, the large volume direct injection method is simpler, more rapid, more precise, and more accurate. It is a viable alternative to SPE, and can be used for the determination of the 42 antibiotics at trace levels in cleaner water bodies, such as surface water, groundwater, and tap water.

随着分析监测技术的不断提高,新型有机污染物已经成为环境领域研究的热点。药品和个人护理品(PPCPs)、消毒副产物(DPBs)、全氟化合物(PFCs)等都属于新型有机污染物。抗生素属于PPCPs中的一种,被广泛地用于人类和动物的治疗,目前在环境中被频繁检出,其种类包括磺胺类、喹诺酮类、四环素、大环内酯类和林可酰胺类^[1]^,检出量一般在ng/L~μg/L级别^[1,2,3,4,5,6]^。目前尚无研究表明该水平的抗生素可对人体造成影响,但是环境中抗生素残留可以诱导环境中耐药基因的产生,使微生物对抗生素产生耐药性^[7]^。目前我国仅在《污水综合排放标准》(GB 8978-1996)和《发酵类制药工业水污染物排放标准》(GB 21903-2008)中规定了部分抗生素的单位产品基准排水量限值,同时国务院颁布的《水污染防治行动计划》明确要求,制药(抗生素、维生素)行业实施绿色酶法生产技术改造。多地也明确要求加强抗生素菌渣监管,加强养殖投入品管理,依法依规限制使用抗生素等化学药品。

现阶段用于抗生素检测的仪器方法包括:液相色谱-串联质谱法、高效液相色谱法、毛细管电泳法等^[5,8-18]^,其中液相色谱-串联质谱法专属性强,灵敏度高,更适用于环境中抗生素的痕量分析。水体中抗生素的前处理方法包括在线固相萃取法^[8,10]^、固相萃取法^[9,11-13]^、分散液液微萃取^[17]^、离子液体膜微萃取法^[18]^,其中固相萃取是最常用于水中抗生素测定的方法。采用固相萃取法虽然可以达到富集浓缩目标物的目的,但在前处理过程复杂且耗时长,而且大部分抗生素属于两性化合物,理化性质差异大,因此用该方法对水中抗生素进行前处理时回收率变化范围很大。例如:Gros等^[19]^采用固相萃取-液相色谱-串联质谱建立了8类53种抗生素的定量分析方法,并对赫罗纳河水进行了检测,回收率为20%~160%;王蕴馨等^[20]^建立了生活饮用水及水源水中3大类10种抗生素的全自动固相萃取-超高效液相色谱-串联质谱(UHPLC-MS/MS)法,回收率为62.4%~119%。

应对突发性应急污染事件,往往样品量需求大,分析时间紧迫,需要一种简单、快速、有效的前处理方法。本方法通过大体积进样提高化合物的灵敏度,前处理过程简单、方便,具备高通量筛查及快速分析的优点。目前采用大体积直接进样法对水中抗生素进行前处理的报道不多,大体积直接进样法大多用于环境水体中农药的处理,进样体积为100 μL^[21,22]^。现有研究中,朱峰等^[23]^建立了一种通过直接进样的方式对水体13种*β*-内酰胺类药物残留进行快速筛查的液相色谱-串联质谱法;Bayen等^[9]^采用液相色谱-电喷雾电离串联质谱法,以小体积直接进样方式分析了地表水和海水中8种特定的抗生素。

本研究采用大体积直接进样方式,建立了超高效液相色谱-串联质谱快速筛查和测定水体中7类42种抗生素的方法。与传统前处理方法相比,本方法具有简单、快速、绿色、准确度高、精密度高、消耗样品量少的优点,为水中抗生素的残留问题提供了简单快速的解决方法。

## 1 实验部分

### 1.1 仪器、试剂与材料

超高效液相色谱-三重四极杆质谱联用仪(SCIEX Triple Quad 6500,美国Sciex公司); 0.22 μm有机相滤膜(美国Waters公司);实验用水为Milli-Q水(美国Bedford公司)。

甲醇、乙腈(色谱纯,美国Merck公司);乙二胺四乙酸二钠(Na_2_EDTA)、甲酸、氨水(色谱纯,德国Sigma-Aldrich公司)。

42种抗生素包括(1)磺胺类(SAs):磺胺氯哒嗪(SCP)、磺胺嘧啶(SDZ)、磺胺间二甲氧嘧啶(SDM)、磺胺甲基嘧啶(SMR)、磺胺二甲嘧啶(SMZ)、磺胺甲噻二唑(SMTZ)、磺胺甲恶唑(SMX)、磺胺噻唑(STZ)、磺胺吡啶(SPD)、磺胺二甲异嘧啶(SM2)、磺胺异恶唑(SIA)、磺胺对甲氧嘧啶(SMT)、磺胺间甲氧嘧(SMM)、磺胺氯吡嗪(SPZ)、磺胺恶喹啉(SQ); (2)林可酰胺类(LINs):克林霉素(CLIN)、林可霉素(LCM); (3)四环素类(TCs):土霉素(OTC)、金霉素(CTC)、四环素(TC)、强力霉素(DOX)、米诺环素(MIC); (4)喹诺酮类(QNs):氧氟沙星(OFX)、诺氟沙星(NOR)、沙拉沙星(SAR)、恩诺沙星(ENR)、环丙沙星(CIP)、洛美沙星(LOM); (5)大环内酯类(MLs):罗红霉素(ROX)、克拉霉素(CLA)、泰乐菌素(TS)、螺旋霉素(SPM)、红霉素(ERY)、阿奇霉素(AZI); (6)头孢类(CEs):头孢噻肟(CTX)、头孢匹林(CP)、头孢克洛(CEC)、头孢唑啉(CFZ)、头孢氨苄(CPX); (7)氯霉素类(CMs):氯霉素(CM)、甲砜霉素(TAP)、氟苯尼考(FFC)。

7种内标包括:磺胺甲基嘧啶-D_4_(SMR-D_4_)、克林霉素-D_3_(CLIN-D_3_)、四环素-D_6_(TC-D_6_)、氧氟沙星-D_3_(OFX-D_3_)、罗红霉素-D_7_(ROX-D_7_)、头孢氨苄-D_5_(CPX-D_5_)、氯霉素-D_5_(CM-D_5_)

所有抗生素标准品均购自百灵威科技有限公司,纯度均大于98.0%。内标均购自德国Dr. Ehrenstorfer公司,纯度均大于98.0%。

### 1.2 标准溶液配制

量取100 mL纯水,加入0.025 g Na_2_EDTA,再用甲酸或氨水调节pH值至6.0~8.0,配制成含有Na_2_EDTA的稀释溶液。

标准品用甲醇(头孢类用50%(v/v)乙腈水溶液)配成1000 μg/mL的标准储备液,于-20 ℃冰箱保存。用甲醇配制1 μg/mL的混合标准溶液,使用时用含Na_2_EDTA的稀释溶剂配制成需要的质量浓度。

7种内标分别用甲醇配制成100 μg/mL的内标储备液;用甲醇进一步稀释配制成SMR-D_4_、CLIN-D_3_和CM-D_5_质量浓度为1.0 ng/mL和TC-D_6_、OFX-D_3_、ROX-D_7_、CPX-D_5_质量浓度为10.0 ng/mL的内标使用液。

### 1.3 样品前处理

量取100 mL水样,经0.22 μm的滤膜过滤后,加入0.025 g Na_2_EDTA,再用甲酸或氨水调节pH值至6.0~8.0。取调节后的水样1.0 mL,加入10 μL内标使用液后,混匀,直接进样。

### 1.4 分析条件

1.4.1 色谱条件

色谱柱:Kinetex C18柱(50 mm×30 mm, 2.6 μm,美国菲罗门公司);流动相A: 0.1%(v/v)甲酸水溶液,流动相B:乙腈;流速:0.4 mL/min;梯度洗脱程序:0~1.5 min, 5%B; 1.5~10.0 min, 5%B~70%B; 10.0~13.0 min, 70%B~90%B; 13.0~14.0 min, 90%B; 14.0~14.2 min, 90%B~5%B; 14.2~15.0 min, 5%B。进样量:100 μL。

1.4.2 质谱条件

采用电喷雾电离(ESI)源,离子源加热温度为500 ℃,检测方式为智能化分时间段-多反应选择离子监测(Schedule-MRM)模式。柱切换:0~1.5 min,切换到废液;1.5~15 min,切换到质谱;正离子/负离子检测。喷雾电压为5500 V/-4500 V;雾化气压力为345 kPa (50 psi);辅助气压力为345 kPa (50 psi);气帘气压力为207 kPa (30 psi)。各化合物的质谱参数见表1。

**表1:** 42种抗生素的质谱参数

Compound	t_R_/min	Precursor ion (m/z)	Product ion (m/z)	Declustering potential/V	Collison energy/eV	IS
Sulfonamides (SAs)						
Sulfachloropyridazine (SCP)	5.2	285.1	156.1^*^	21	20.3	SMR-D_4_
			92.0	21	39.2	
Sulfadiazine (SDZ)	3.1	251.1	156.0^*^	40	22.0	
			92.0	40	32.1	
Sulfadimethoxine (SDM)	6.3	311.1	156.1^*^	120	28.0	
			108.2	140	47.0	
Sulfamerazine (SMR)	4.0	265.2	172.1^*^	70	23.0	
			156.1	70	23.0	
Sulfamethazine (SMZ)	4.6	279.2	186.1^*^	45	24.0	
			124.2	45	33.0	
Sulfamethizole (SMTZ)	4.6	271.0	156.2^*^	30	19.3	
			108.0	30	35.0	
Sulfamethoxazole (SMX)	5.5	254.0	156.1^*^	49	23.0	
			108.0	49	31.0	
Sulfathiazole (STZ)	3.7	256.1	156.1^*^	38	21.0	
			108.1	38	31.0	
Sulfapyridine (SPD)	3.8	250.3	184.3^*^	60	25.0	
			156.1	60	23.0	
Sulfisomidine (SM2)	3.0	279.2	186.1^*^	70	23.0	
			124.4	70	30.0	
Sulfisoxazole (SIA)	5.7	268.2	156.0^*^	60	20.0	
			113.3	60	19.0	
Sulfameter (SMT)	4.7	281.2	215.3^*^	60	25.0	
			156.0	60	25.0	
Sulfamonomethoxine (SMM)	5.1	281.1	156.1^*^	100	25.0	
			215.1	100	25.0	
Sulfachloropyrazine (SPZ)	6.1	285.2	156.1^*^	120	22.0	
			92.1	120	37.0	
Sulfaquinoxaline (SQ)	6.3	301.1	156.0^*^	120	22.0	
			92.2	120	40.0	
Lincosamides (LINs)						
Clindamycin (CLIN)	5.6	425.3	377.2^*^	80	27.0	CLIN-D_3_
			126.2	80	34.0	
Lincomycin (LCM)	3.9	407.2	126.2^*^	120	35.0	
			359.2	120	25.0	
Tetracyclines (TCs)						
Oxytetracycline (OTC)	4.4	461.3	443.2^*^	100	19.0	TC-D_6_
			426.2	100	27.0	
Chlortetracycline (CTC)	5.2	479.2	462.0^*^	120	24.0	
			444.1	120	31.0	
Tetracycline (TC)	4.5	445.2	427.3^*^	120	19.0	
			410.3	120	27.0	
Doxycycline (DOX)	4.7	445.2	428.1^*^	140	27.0	
			410.2	140	34.0	
Minocycline (MIC)	4.1	458.2	441.2^*^	130	25.0	
			352.2	130	40.0	
Quinolones (QNs)						
Ofloxacin (OFX)	4.5	362.3	318.0^*^	140	26.0	OFX-D_3_
			261.0	140	38.0	
Compound	t_R_/min	Precursor ion (m/z)	Product ion (m/z)	Declustering potential/V	Collison energy/eV	IS
Norfloxacin (NOR)	4.5	320.1	302.3^*^	140	27.0	
			276.1	140	24.0	
Sarafloxacin (SAR)	5.2	386.2	342.3^*^	100	25.0	
			299.0	100	38.0	
Enrofloxacin (ENR)	4.9	360.0	316.2^*^	100	27.0	
			245.2	100	36.0	
Ciprofloxacin (CIP)	4.6	332.1	314.0^*^	90	31.0	
			288.3	90	25.0	
Lomefloxacin (LOM)	4.7	352.0	265.2^*^	100	32.0	
			308.1	100	23.0	
Macrolides (MLs)						
Roxithromycin (ROX)	7.4	837.3	679.2^*^	100	30.0	ROX-D_7_
			158.1	100	38.0	
Clarithromycin (CLA)	7.3	748.3	590.4^*^	120	27.0	
			158.2	120	33.0	
Tylosin (TS)	6.8	916.5	772.4^*^	130	41.0	
			174.1	130	47.0	
Spiramycin (SPM)	5.5	843.4	174.0^*^	120	45.0	
			540.0	120	42.0	
Erythromycin (ERY)	6.5	734.3	576.2^*^	80	27.0	
			158.3	80	35.0	
Azithromycin (AZI)	5.5	749.7	591.5^*^	160	40.0	
			573.6	160	47.0	
Cephalosporins (CEs)						
Cefotaxime (CTX)	4.5	456.0	396.0^*^	60	15.0	CE-D_5_
			324.0	60	19.0	
Cephapirin (CP)	3.6	424.2	292.2^*^	60	21.0	
			152.0	60	29.0	
Cefaclor (CEC)	3.8	368.0	174.2^*^	29	20.0	
			106.1	29	39.0	
Cefazolin (CFZ)	4.9	455.1	323.0^*^	37	15.0	
			156.0	37	20.0	
Cephalexin (CPX)	4.2	348.2	174.0^*^	80	23.0	
			158.2	80	15.0	
Chloramphenicols (CMs)						
Chloramphenicol (CM)	4.4	321.0	257.1^*^	-40	-15.0	CM-D_5_
			152.0	-40	-24.0	
Thiamphenicol (TAP)	3.2	354.0	290.2^*^	-50	-17.0	
			185.1	-50	-27.0	
Florfenicol (FFC)	4.1	356.0	336.0^*^	-40	-13.0	
			185.0	-40	-26.0	
IS						
Sulfamerazine-D_4_(SMR-D_4)_	4.0	269.2	172.1	70	23.0	
Cephalexin-D_5_(CPX-D_5_)	4.1	353.0	179.0	80	23.0	
Ofloxacin-D_3_(OFX-D_3_)	4.4	365.2	321.1	140	26.0	
Tetracycline-D_6_(TC-D_6_)	4.6	451.4	416.2	120	27.0	
Clindamycin-D_3_(CLIN-D_3_)	5.5	428.0	129.0	80	34.0	
Roxithromycin-D_7_(ROX-D_7_)	7.4	844.3	686.2	100	30.0	
Chloramphenicol-D_5_(CM-D_5_)	4.4	326.0	157.0	-24	-40.0	

* Quantitative ion.

## 2 结果与讨论

### 2.1 质谱条件的优化

在ESI源和正、负离子扫描模式下,配制42种抗生素及其内标混合溶液(100 μg/L),采用流动注射进入质谱进行扫描,确定最佳去簇电压、碰撞能量及各化合物的母离子和子离子等质谱参数,优化后的质谱条件见表1。

2.1.1 质谱离子源温度的选择

水样经过固相萃取富集浓缩后,一般用有机溶剂定容并进样分析,因此对离子源温度要求不高(250 ~ 300 ℃),但由于本方法采用大体积水溶液直接进样,水溶液的雾化效率不如有机溶剂,因此质谱离子源的雾化温度对样品离子化效率至关重要。本研究比较了250、400和500 ℃这3种不同离子源温度下各目标物的响应情况(见图1)。结果表明,在仪器推荐的温度范围内,随着离子源温度的升高,各类化合物的响应也呈现明显的增强,这是由于大体积水溶液的进样需要更高的温度才能使之更好地完成离子化。因此,实验选择500 ℃作为离子源的温度。

**图1:**
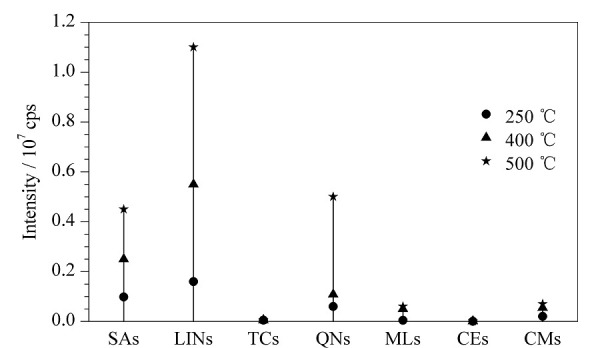
不同离子源温度下各类抗生素的响应值

2.1.2 采集模式的选择

本方法还采用了智能化分时间段-多反应选择离子监测的采集模式,相比常规的多反应选择离子监测模式,该采集方式通过对一个分析测试周期的时间进行合理分段,避免了常规的全分析周期全离子通道扫描,不同时间段仅扫描色谱出峰的离子通道,从而增加了每个目标物离子通道的扫描时间,进一步提高了各目标物的响应,方法灵敏度提高2~4倍。图2为42种抗生素标准溶液(0.5 μg/L)的MRM色谱图。

**图2:**
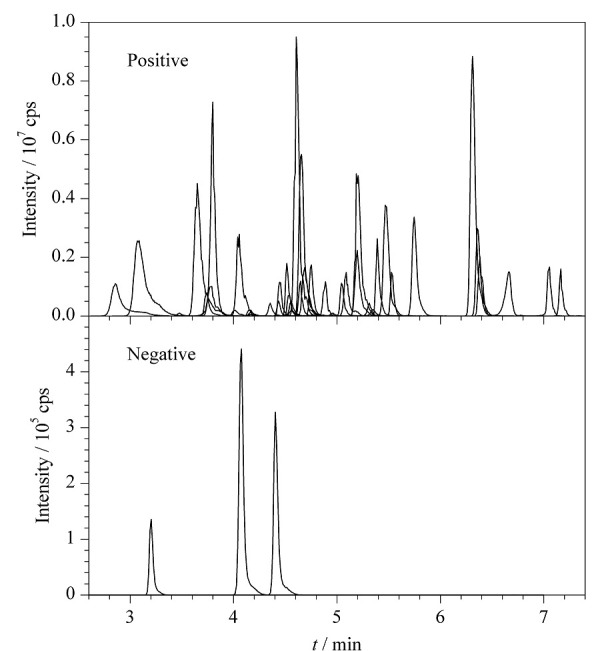
42种抗生素(0.5 μg/L)在正离子和负离子 模式下的总离子流图

### 2.2 Na_2_EDTA的添加对回收率的影响

四环素类和喹诺酮类抗生素易与水中金属形成稳定的络合物从而影响测定结果,因此本方法考察了实际水样中Na_2_EDTA的加入对各类抗生素回收率的影响(见图3)。研究发现,未添加Na_2_EDTA时,四环素类回收率仅为0%~15%,喹诺酮类回收率为35%~50%。添加Na_2_EDTA后,四环素类回收率显著提高为94%~105%,但喹诺酮类回收率并未有提高,同时发现它们的质谱响应比未添加Na_2_EDTA时降低了近50~100倍,这是因为Na_2_EDTA加入后水样呈偏酸性(pH≈5)。因此添加Na_2_EDTA后需要对水样pH值进行调节从而使喹诺酮类的质谱响应回升。

**图3:**
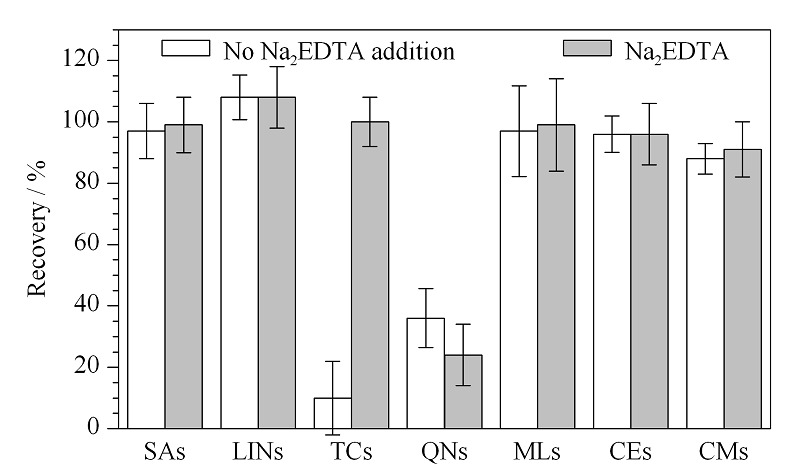
Na_2_EDTA的添加对抗生素回收率的影响(*n*=3)

### 2.3 pH的优化

头孢类抗生素的*β*-内酰胺环在酸、碱性条件下易发生水解;四环素类抗生素在碱性条件下会发生差向异构化和降解反应;大环内酯类在碱性条件下易发生开环反应,因此本方法考察了溶液在中性条件下实际水样的回收率。对比了添加Na_2_EDTA,并将pH调节至6.0、6.5、7.0、7.5、8.0后6大类抗生素的回收率(见图4)。结果可见,喹诺酮类的回收率得到了改善,且各大类抗生素的回收率为80.1%~125%,均能满足实验要求。因此本研究选择水样中加入Na_2_EDTA后并将水溶液pH值调节至6.0~8.0进行测定分析。

**图4:**
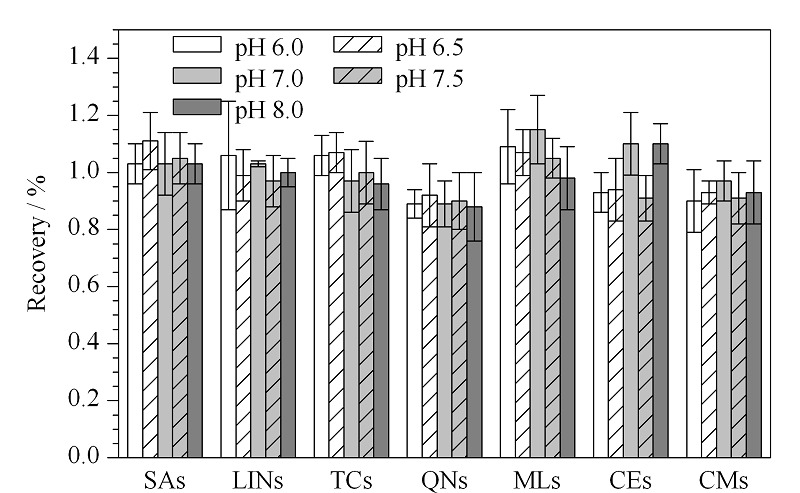
pH值对抗生素回收率的影响(*n*=3)

### 2.4 与固相萃取法的比较

分别考察了采用大体积直接进样法和固相萃取法时水体中抗生素的回收率。不同种类的抗生素由于具有不同的酸碱性,其p*K*_a_值为2.1~9.0,因此前处理时需要调节不同的pH值使之更好地被固相萃取小柱吸附萃取。根据文献^[24,25]^及以往的经验,Oasis HLB柱和MCX柱适用于分析不同种类的抗生素。因此将本方法与采用Oasis HLB柱和MCX柱的固相萃取法的回收率进行比较,具体流程见图5。结果如图6所示,大体积直接进样法对大多数抗生素的回收率优于固相萃取法。本方法没有经过复杂的前处理过程,目标化合物损失少,更适合于水体中化学性质各异的抗生素的检测。

**图5:**
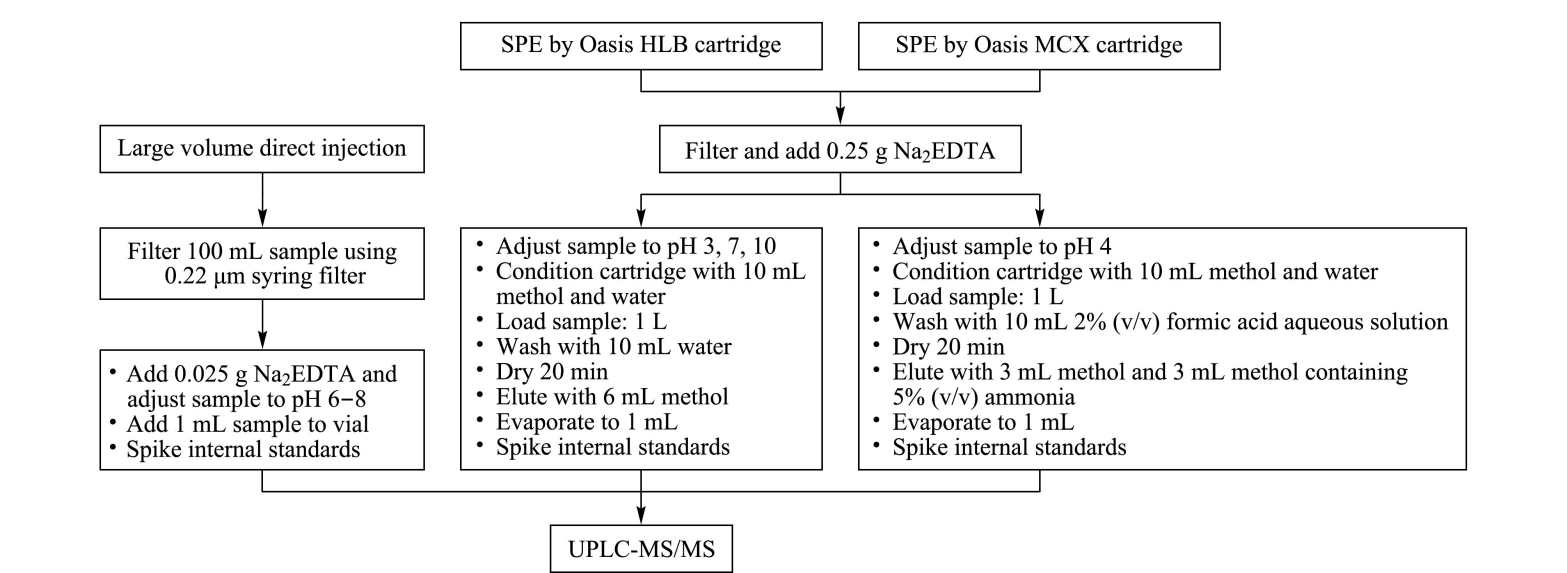
3种前处理方法的流程图

**图6:**
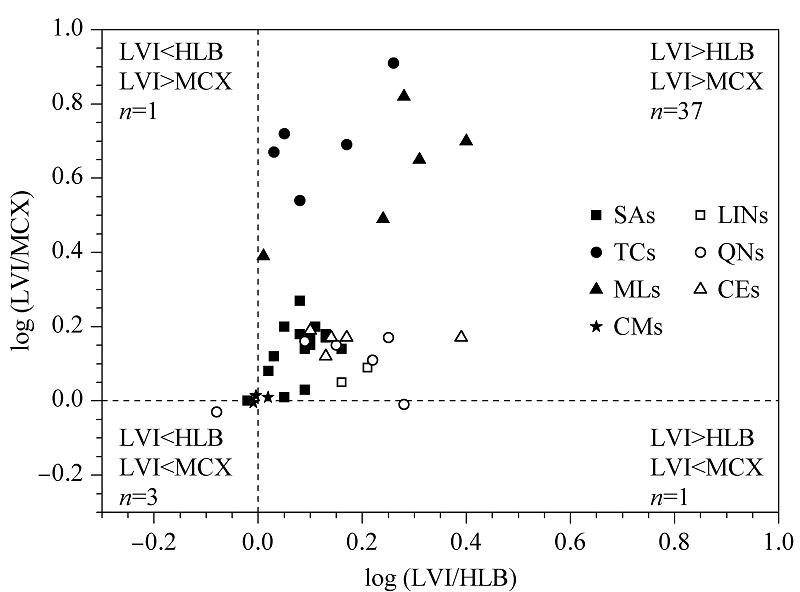
采用大体积直接进样法与固相萃取法时42种 抗生素回收率的比较

### 2.5 方法学验证

2.5.1 线性范围与方法检出限

配制每种化合物的系列混合标准溶液,按确定的分析条件进行测定,以各物质的质量浓度(ng/L)为横坐标*X*,以其对应的峰面积与内标峰面积比值为纵坐标(*Y*),绘制标准曲线。实验结果显示,42种抗生素在各自的线性范围内线性良好(见附表1,详见http://www.chrom-China.com)。

根据HJ 168-2020方法检出限测定要求,将42种低浓度抗生素标准溶液添加至超纯水中,按照样品分析过程平行测定7份。结果表明,42种抗生素的方法检出限为0.015 ~ 3.561 ng/L(见附表1)。方法灵敏度高,完全能够满足环境水体中该抗生素检测要求。

2.5.2 准确度与精密度

分别于空白纯水和地表水中加入质量浓度范围在50~500 ng/L之间的低、高两种水平的标准混合工作液,每个水平各平行测定3次,考察其回收率及相对标准偏差,方法的回收率为80.1%~125%,相对标准偏差为0.8%~12.2%(见附表2)。

### 2.6 与其他方法比较

2.6.1 检出限

抗生素在环境水体中一般为痕量水平,且大体积直接进样法未有浓缩富集的步骤,化合物的质谱响应是通过提高进样量和优化质谱条件来提高的,因此灵敏度的高低是该方法能否直接应用于环境水体中抗生素残留检测的关键。本研究将大体积直接进样法的方法检出限与现有的抗生素标准及文献报道中的方法检出限进行比较(见表2),表明本方法可以满足标准方法的要求,大多数化合物的方法检出限低于文献报道,说明直接进样法的灵敏度高,符合痕量检测的需求。

**表2:** 本方法与标准方法及其他文献方法检出限的比较

Compound	MDLs/(ng/L)		
	This method	Standards	Literatures
SAs	0.015-0.349	0.3-1.2^[26]^, 70-100^[27]^, 1.0^[28]^, 0.5-10^[29]^	0.19-1.57^[30]^, 5.3-23.9^[31]^, 0.41-5.49^[19]^, 0.721^[32]^
LINs	0.022-0.060	0.8^[26]^	0.48-6.04^[19]^, 1.01^[30]^
TCs	1.110-3.201	1.2-51^[26]^	4.72-11.23^[19]^, 0.63-1.53^[30]^
QNs	0.582-2.004	1.8-170^[26]^, 30^[27]^, 3.0^[28]^	0.54-13.55^[19]^, 0.30-1.09^[30]^, 33.3-35.3^[32]^
MLs	0.312-3.561	0.2-13^[26]^, 70^[27]^, 7.0^[28]^	0.31-3.99^[19]^, 0.16-0.52^[30]^, 4.17^[32]^
CEs	0.122-3.064	10^[26]^, 70^[27]^	0.77-13.37^[19]^, 1.15^[30]^, 0.06-2.66^[33]^
CMs	0.172-0.231	/	0.65-0.77^[30]^, 1.13^[33]^

2.6.2 回收率

将本方法的回收率与文献报道的固相萃取法的回收率进行比较。如表3所示,经过固相萃取的抗生素回收率差异较大,这是由于每种抗生素的理化性质差异大,固相萃取法难以涵盖全面,大体积直接进样法可以弥补固相萃取法的不足,兼顾各类抗生素的回收率。大体积直接进样法消耗的样品量少和有机溶剂少,更加绿色环保;无需前处理设备、玻璃器皿的重复使用减少了残留的可能性;前处理过程大大缩短可以实现快速检测,因此该方法适用于高通量的样品测定与快速分析。

**表3:** 与其他文献方法的回收率比较

Compound	Recoveries/%	
	Large volume direct injection (this study)	Solid phase extraction (literature)
SAs	80.2-125	2-134^[30]^, 32-104^[19]^
LINs	94.0-110	62-112^[19]^, 89-141^[30]^
TCs	95.6-122	50.2-94.6^[5]^, 52-118^[19]^, 37-218^[30]^, 50^[34]^
QNs	80.1-108	47-126^[19]^, 9-173^[30]^, 75^[35]^, 56.6-74.6^[36]^, 7.4-90.2^[18]^
MLs	81.7-116	54.2-98.4^[19]^, 75-162^[30]^, 63.1-69.6^[36]^, 51-104^[37]^, 36.0-57.8^[32]^
CEs	86.9-115	129-131^[19]^, 15-112^[33]^, 65-101^[38]^, 91^[39]^
CM	83.1-101	53-81^[30]^, 73^[35]^, 108-128^[33]^, 57-68^[40]^

### 2.7 实际样品的分析

应用本方法对长江流域的10个点位的水源地水及江苏省某市的5个点位的末梢水开展42种抗生素监测,共检出了12种抗生素,总含量范围为ND~80.3 ng/L(见表4)。磺胺类、林可酰胺类、大环内酯类和氯霉素类普遍检出,在水源地水中这4大类的检出率为100%; 12种检出的抗生素中含量最高的为氟苯尼考,范围为24.8~33.6 ng/L,其次是林可霉素,范围为3.83~13.8 ng/L,其余种类的抗生素在所有点位均未被检出。

**表4:** 实际水样中抗生素的阳性检测结果

Site	Contents/(ng/L)											
	SCP	SDZ	SMZ	SMX	SM2	ROX	CLA	ERY	CLIN	LCM	TAP	FFC
S1	3.82	4.02	0.66	4.78	0.56	2.70	2.18	2.71	2.45	12.4	6.80	31.4
S2	3.40	3.03	0.64	4.43	0.82	2.92	2.38	2.81	2.57	11.5	6.30	29.6
S3	3.33	2.60	0.46	3.91	0.58	2.74	2.36	2.67	2.09	11.2	5.18	23.9
S4	3.83	2.57	0.66	4.44	0.69	3.24	2.53	2.58	3.38	13.7	4.92	28.1
S5	3.54	3.00	0.56	4.53	0.65	2.84	2.79	2.95	3.55	12.6	4.88	27.1
S6	3.84	2.38	0.57	4.98	0.66	3.36	2.71	2.60	2.92	12.5	5.35	25.5
S7	3.58	6.56	0.52	4.09	0.59	2.69	2.25	2.49	2.57	13.1	4.53	24.8
S8	3.20	4.46	0.65	3.76	0.62	2.94	5.03	2.21	2.48	13.8	7.57	33.6
S9	3.82	2.89	0.50	4.17	0.59	4.02	2.64	2.26	2.78	13.0	6.68	29.3
S10	3.06	0.22	ND	3.37	ND	ND	ND	ND	1.22	3.83	5.74	31.7
G1	ND	ND	ND	ND	ND	ND	ND	ND	ND	ND	ND	ND
G2	ND	ND	ND	ND	ND	ND	ND	ND	ND	ND	ND	ND
G3	ND	ND	ND	ND	ND	ND	ND	ND	ND	ND	ND	ND
G4	ND	ND	ND	ND	ND	ND	ND	ND	ND	ND	ND	ND
G5	ND	ND	ND	ND	ND	ND	ND	ND	ND	ND	ND	ND

S1-S10: drinking water sources; G1-G5: tap water; ND: not detected.

## 3 结论

建立了大体积进样直接检测环境水体中7大类42种抗生素的超高效液相色谱-串联质谱法,与固相萃取方法相比大大节省方法开发的时间,提高工作效率,具有简单、快速、精密度高、准确度高、灵敏度高、样品消耗量小的优点,为地表水、地下水、末梢水等较为洁净的水体中抗生素的检测提供了简单、快速、可靠的解决方案,同时也为高通量的快速筛查提供了优势。
